# SPHK1 promotes HNSCC immune evasion by regulating the MMP1-PD-L1 axis

**DOI:** 10.7150/thno.102390

**Published:** 2024-10-28

**Authors:** Qi Fang, Xiao Chen, Fei Cao, Pengfei Xu, Zheng Zhao, Roubin Lin, Di Wu, Wuguo Deng, Xuekui Liu

**Affiliations:** 1 Department of Head and Neck Surgery, Sun Yat-Sen University Cancer Center, 651 Dongfeng East Road, Guangzhou 510060, Guangdong, People's Republic of China.; 2 State Key Laboratory of Oncology in South China, 651 Dongfeng East Road, Guangzhou 510060, Guangdong, People's Republic of China.; 3 State Key Laboratory of Oncology in South China, Guangdong Provincial Clinical Research Center for Cancer, Sun Yat-sen University Cancer Center, Guangzhou 510060, China.; 4 The General Surgery Department of The Second Hospital of Anhui Medical University, Hefei, China.; 5 Anhui Medical University, Hefei, China.; 6 Sun Yat-Sen University, Guangzhou, China.

**Keywords:** HNSCC, SPHK1, MMP1, PD-L1, PD-1.

## Abstract

**Rationale:** Immune checkpoint inhibitors (ICIs) have demonstrated significant efficacy against head and neck squamous cell carcinoma (HNSCC), but their overall response rate (ORR) remains limited. Previous studies have highlighted the crucial role of sphingosine kinases (SPHKs) in the tumor microenvironment (TME); however, their function in immunotherapy remains unclear.

**Methods:** We conducted comprehensive bioinformatics analysis, functional studies, and clinical validation, to investigate the role of SPHK1 in the immunology of HNSCC.

**Results:** Functionally, SPHK1 significantly promoted tumor growth by inhibiting anti-tumor immunity in immune-competent HNSCC mouse models and tumor-T cell co-cultures. Mechanistic analysis revealed that SPHK1 regulated matrix metalloproteinase-1 (MMP1) expression via the MAPK1 pathway, which subsequently influenced tumor programmed cell death ligand 1 (PD-L1) expression. Furthermore, SPHK1 and MMP1 could predict the efficacy of programmed cell death 1 monoclonal antibody (PD-1 mAb) immunotherapy in HNSCC and were independent risk factors for survival in patients with HNSCC.

**Conclusion:** Our study reveals a novel role for SPHK1 in mediating immune evasion in HNSCC through the regulation of the MMP1-PD-L1 axis. We identified SPHK1 and MMP1 as predictive biomarkers for the therapeutic response to PD-1 mAb and provided new therapeutic targets for patients with HNSCC.

## Introduction

Head and neck squamous cell carcinoma (HNSCC) is the most common type of head and neck cancer, accounting for approximately 90% of all head and neck cancers globally 1. HNSCC is the eighth most prevalent cancer worldwide, with approximately 870,000 new cases and 440,000 deaths recorded in 2020. The incidence of HNSCC continues to rise, and it is projected to increase by 30% by 2030 (i.e., 1.08 million new cases annually) 2-3. Advances in the understanding of the immune characteristics and immunogenicity of HNSCC have led to the wide application of a range of immunotherapies in clinical practice 4-5. Therefore, identifying the molecular mechanisms underlying anti-tumor immunity in HNSCC cells can lay the foundation for expanding immunotherapy strategies.

Sphingosine kinases (SPHKs) are crucial rate-limiting enzymes with two subtypes, SPHK1 and SPHK2, which catalyze the phosphorylation of sphingosine (SPH) to sphingosine-1-phosphate (S1P) 6-7. Despite sharing the same substrate and highly similar amino acid sequences, SPHK1 and SPHK2 exhibit significant differences in their expression levels and subcellular localization 8-9. SPHK1 is localized in the cytoplasm of cells across various organs, whereas SPHK2 is predominantly distributed in the nucleus and organelles 10. Cumulative evidence to date indicates that S1P generated by SPHK1 can be transported to the cell membrane via G protein-coupled receptors and is involved in the formation of the tumor microenvironment (TME) 11. Additionally, SPHK1 plays a pivotal role in the “inside-out” signaling transport of S1P 12. Prior evidence also suggests that sphingolipids participate in both normal biological processes and those within cancer cells 13. SPHKs serve as key mediators of “sphingolipid regulators” 14. Upregulation of SPHK1 expression has been observed in various cancers, including gliomas, lung cancer, colon cancer, and breast cancer, and is associated with poor survival outcomes in glioma, lung cancer, and breast cancer 15-16. Previous studies have found that high SPHK1 expression can promote tumor migration, invasion, and angiogenesis through multiple mechanisms, such as the SPHK1/miR-144-3p/FN1 and SPHK1/p-PAK axes 17. Therefore, targeting SPHK1 may represent a crucial strategy for inhibiting cancer progression. Recent research has revealed the role of SPHK1 in cancer progression 18-19. However, the specific mechanisms of SPHK1 involvement in tumor immune evasion remain unclear.

Matrix metalloproteinases (MMPs) are a class of zinc-dependent proteases that degrade or proteolytically cleave components of the extracellular matrix 20-21. Matrix metalloproteinase-1 (MMP1), a member of the MMP family, is associated with poor survival outcomes in breast, prostate, and gastric cancers because of its increased expression 22-23. Previous studies have found that high expression of MMP1 can lead to tumor migration, invasion, and angiogenesis through various mechanisms, including the inhibition of breast cancer metastasis by downregulation of MMP1 via bone morphogenetic protein 6 (BMP-6) and the promotion of glioma cell invasion by MMP1 through the mitogen-activated protein kinase (MAPK) pathway 24-25. However, the relationship between MMP1 and programmed cell death ligand 1 (PD-L1), as well as the role of MMP1 in the TME, remains unclear.

Through comprehensive analysis, SPHK1 has been shown to be associated with the immune characteristics of various cancers and positively correlated with PD-L1 26. PD-L1 serves as a ligand within the B7 family 27. In HNSCC, PD-L1 expression can be induced and upregulated through both innate and adaptive mechanisms 28. Abnormally high PD-L1 expression in tumor cells is a crucial factor in immune tolerance and immune surveillance evasion 29. When PD-L1 on tumor cells binds to its cognate inhibitory receptor PD-1 expressed on tumor-infiltrating lymphocytes (TILs), the inhibitory signaling pathway induced by PD-L1 negatively regulates T-cell activation and proliferation, promoting T cell exhaustion 30. The advancement of immune checkpoint inhibitors (ICIs) has ushered in a new era of cancer treatment, with PD-1 blockade therapy emerging as a breakthrough 31-32.

However, due to the complexity of the TME and inter-individual differences, the therapeutic effects of these drugs on solid malignancies are not ideal 33-35. Consequently, exploring the mechanisms of resistance to ICIs must be explored.

The aim of this study was to evaluate the role of SPHK1 in anti-tumor immunity, reveal the novel molecular mechanism of PD-L1 regulation through MMP1, and further explore the clinical significance of the SPHK1-MMP1 axis in HNSCC immunotherapy.

## Results

### Relationship between SPHK1 expression and the immune characteristics of HNSCC

**Figure 1:**
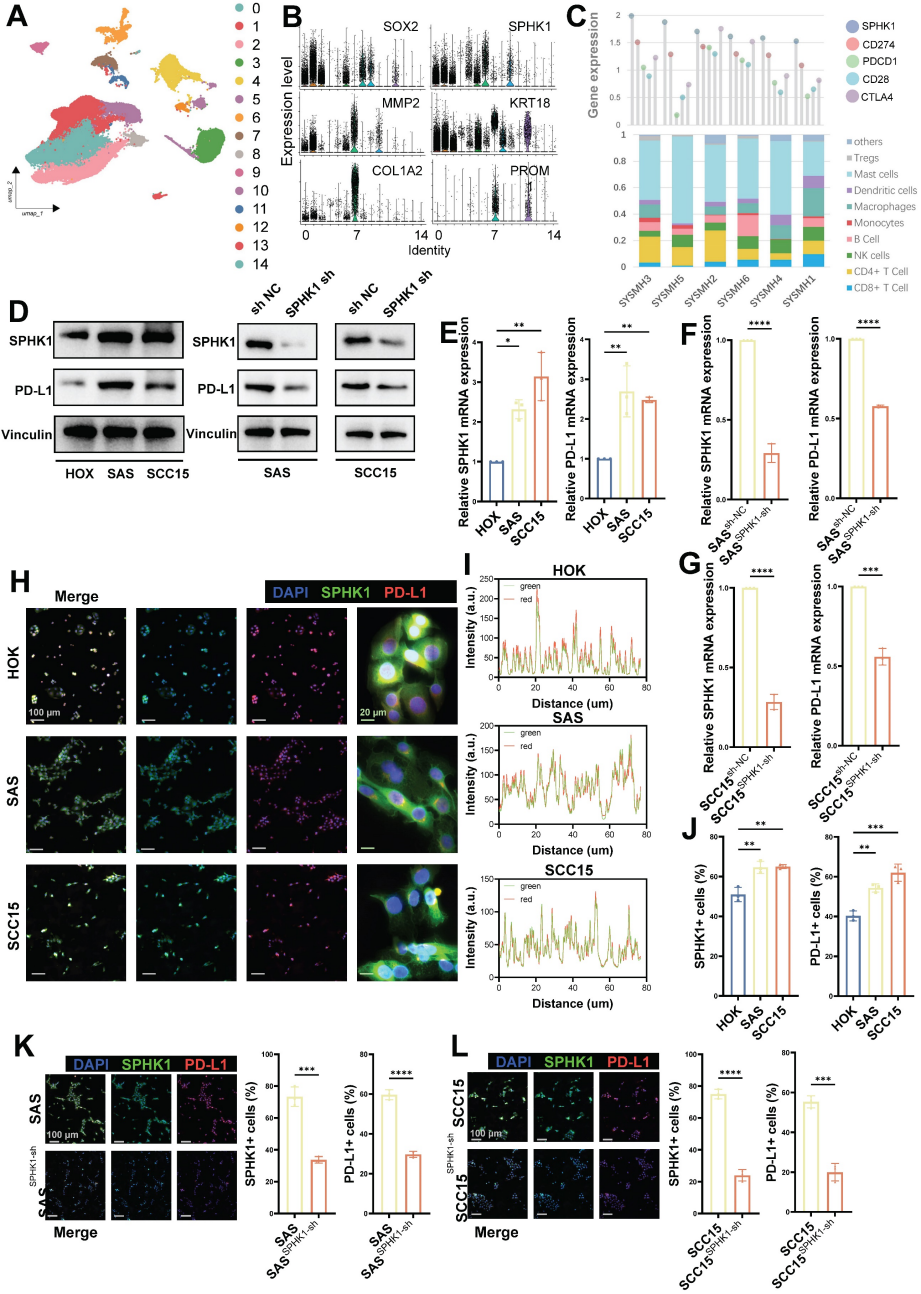
Associations between SPHK1 and tumor-infiltrating lymphocytes and inhibitory biomarkers. **A.** Subgroups of cells in six patients with oral squamous cell carcinoma. **B.** Expression of SPHK1 in different subtypes of squamous cells from the oral squamous cell carcinoma model. **C.** Correlation between the proportion of immune -infiltrating cells and immune checkpoint inhibitor levels with the level of SPHK1 in six patients with oral squamous carcinoma. **D.** Western blot analysis demonstrating both the protein levels of SPHK1 and PD-L1 in the normal oral epithelial keratinocyte cell line HOK and the HNSCC cell lines SAS and SCC15, as well as the effect of SPHK1 knockdown on PD-L1 protein levels in the HNSCC cell lines SAS and SCC15. **E-F.** qPCR analyses reveal the mRNA levels of SPHK1 and PD-L1 in both the normal oral epithelial keratinocyte cell line HOK and the HNSCC cell lines SAS and SCC15, as well as the impact of SPHK1 knockdown on PD-L1 mRNA levels in the HNSCC cell lines SAS and SCC15. **H-J.** Multiplex immunofluorescence staining was performed in HOK, SAS, and SCC15 cells to analyze the co-localization of SPHK1 and PD-L1 (I) and to quantify the ratio of cells expressing both SPHK1^+^ and PD-L1^+^ markers (J). **K-L.** Multiple immunofluorescence staining shows the effect of SPHK1 knockdown on the proportion of PD-L1+ cells in SAS and SCC15 cells. Quantitative data derived from immunofluorescence analysis are presented on the right-hand side of the figure. Error bars represent the mean ± SD of three independent experiments. Statistical significance was determined using one-way ANOVA with Tukey's multiple comparisons test. *P < 0.05; **P < 0.01; ***P < 0.001. NS: No significance, PD-L1: Programed cell death ligand 1.

To investigate the role of SPHK1 in HNSCC cells, we analyzed a public single-cell dataset (GSE172577; www.ncbi.nlm.nih.gov/geo) comprising primary tumor tissues from six untreated patients with oral squamous cell carcinoma (39,064 cells; Figure 1A). Single-cell analysis revealed high expression of SPHK1 in tumor cells (SOX2, KRT18) and cancer-associated fibroblasts (COL1A2 and MMP2) and low expression in cancer stem cells (PROM1) (Figure 1B). After extracting scRNA_count information from the six patients, correlation analysis of immune checkpoints showed that SPHK1 was positively correlated with PD-L1, PD-1, and CD28 but negatively correlated with CTLA4 (Figure 1C). The markers for immune cells provided on the CIBERSORTx website (https://cibersortx.stanford.edu/) were used to calculate the proportion of immune-infiltrating cells in six patients with oral squamous carcinoma and correlate them with SPHK1 levels. The results showed that SPHK1 was negatively correlated with the abundance of CD8^+^ T cells and NK cells in patients (Figures 1C and S1A).

The same results were observed for the relationship between SPHK1 and immune checkpoints and immune-infiltrating cells in TCGA (The Cancer Genome Atlas) dataset of patients with HNSCC (Figure S1B-C), and SPHK1 expression was inversely associated with overall survival (OS) and disease-free survival (DFS) (Figure S1D). Further experiments demonstrated that compared to the normal oral keratinocyte cell line HOK, the mRNA and protein levels of SPHK1 and PD-L1 were both elevated in the HNSCC cell lines SAS and SCC15 (Figure 1D, E). Subsequently, stable HNSCC cell lines SAS and SCC15 with knocked-down SPHK1 were established via viral transfection. The results revealed that knocking down SPHK1 reduced PD-L1 mRNA and protein expression in HNSCC (Figure 1D, F). Immunofluorescence staining confirmed the co-localization of SPHK1 and PD-L1 in the HNSCC cell lines SAS and SCC15 (Figure 1H, I), with a higher proportion of SPHK1^+^/PD-L1^+^ cells compared to the normal oral keratinocyte cell line HOK (Figure 1J). When immunofluorescence staining was performed on SPHK1-depleted stable SAS and SCC15 cells, the proportion of PD-L1^+^ cells was also downregulated (Figure 1K, L). These findings support that PD-L1 expression is regulated by SPHK1 in HNSCC cells and is negatively correlated with immune-infiltrating cells such as CD8^+^ and NK cells.

### MMP1 regulates PD-L1 expression in HNSCC as a downstream target of SPHK1

**Figure 2:**
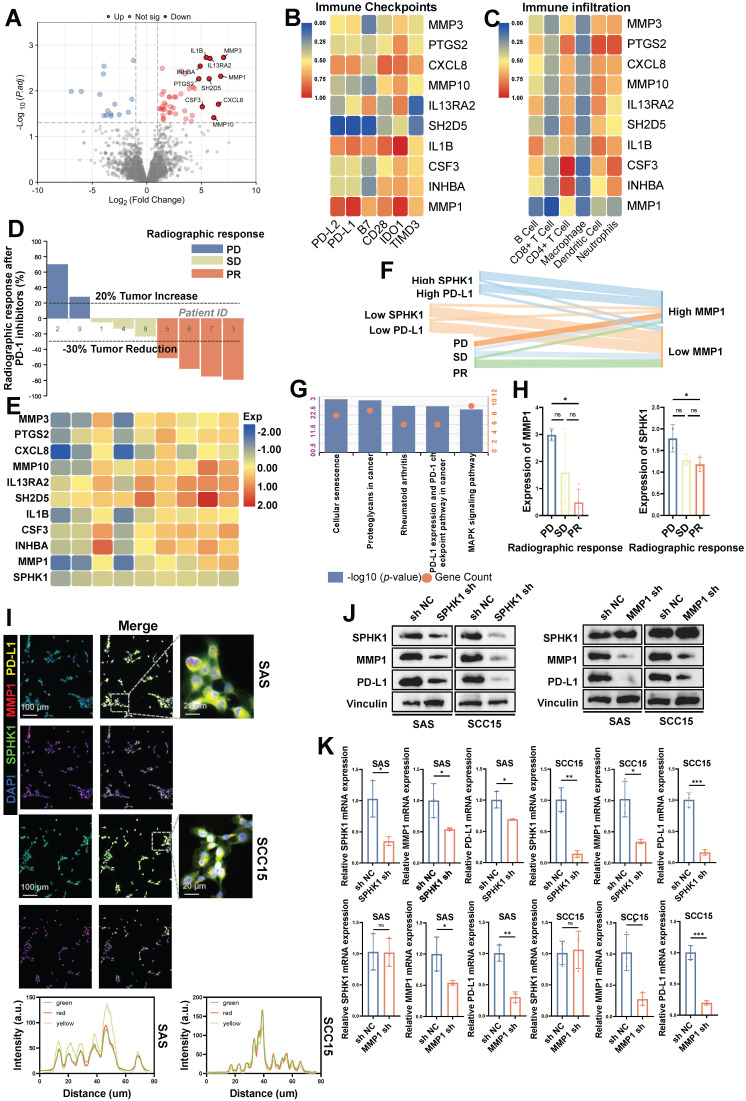
MMP1 acts as a downstream target of SPHK1 to regulate PD-L1 in HNSCC. **A.** Fresh tumor tissues from nine patients with HNSCC before immunotherapy were analyzed by RNA sequencing, and differential analysis was performed based on SPHK1 expression levels. **B-C.** Correlation analysis of the proportion of immune-infiltrating cells and immune checkpoint levels according to the ten candidate genes in nine patients with HNSCC.** D.** Evaluation of the radiographic response in nine patients with HNSCC after 2 weeks of treatment with PD-1 inhibitors. **E.** Association of ten candidate genes and SPHK1 with radiological response after 2 weeks of PD-1 inhibitor therapy. **F.** Distribution of SPHK1, MMP1, and PD-L1 levels among patients with different immunotherapy efficacies.** G.** Enrichment analysis of aberrantly expressed genes in the MMP1 expression differential group by KEGG. **H.** Comparison of SPHK1 and MMP1 expression in patients with different radiographic responses. **I.** Co-localization analysis of SPHK1, MMP1, and PD-L1 was detected by immunofluorescence staining in the HNSCC cell lines SAS and SCC15.** J.** Western blotting showed the effect of SPHK1 knockdown in the HNSCC cell lines SAS and SCC15 on MMP1 and PD-L1 protein levels. **K.** qPCR analyses showing the effect of SPHK1 knockdown on MMP1 and PD-L1 mRNA expression in SAS and SCC15 HNSCC cells. Error bars represent the mean ± SD of three independent experiments. Statistical significance was determined using one-way ANOVA with Tukey's multiple comparisons test. *P < 0.05; **P < 0.01; ***P < 0.001. NS: No significance, PD-L1: Programmed cell death ligand 1.

Subsequently, to explore the downstream signaling pathway regulating PD-L1 expression induced by SPHK1 in HNSCC, we selected fresh tumor tissues from nine patients with tongue squamous cell carcinoma before immunotherapy for RNA sequencing (RNA-seq) analysis. Among these, there were four cases in the SPHK1 high-expression group and five cases in the low-expression SPHK1 group. Among all upregulated genes associated with high SPHK1 expression, ten candidate genes (MMP3, PTGS2, CXCL8, MMP10, IL13RA2, SH2D5, IL1B, CSF3, INHBA, and MMP1) were identified based on the fold change of differentially expressed genes (Figure 2A). Further analysis of the correlation between the candidate genes and the percentage of immune cell infiltration and the expression level of immune checkpoints in the 9 patients revealed that only MMP1 was negatively correlated with PD-L1 and PD-L2 (P < 0.001) (Figure 2B), and MMP1 was also negatively correlated with the abundance of infiltrating immune cells, including CD8^+^ T cells (P < 0.001) (Figure 2C). Through the analysis of the imaging efficacy assessment of nine patients 2 weeks after PD-1 inhibitor treatment, it was found that both SPHK1 and MMP1 were negatively correlated with the efficacy of PD-1 inhibitors (Figure 2D-E), and the distribution of SPHK1, MMP1, and PD-L1 was consistent among patients with different immunotherapy efficacies (Figure 2F). The expression levels of SPHK1 and MMP1 in patients achieving PD in the imaging efficacy assessment 2 weeks after PD-1 inhibitor treatment were higher than those in patients achieving PR, with statistically significant differences (Figure 2H). KEGG enrichment analysis of abnormally expressed genes in the MMP1 differentially expressed group revealed that MMP1 was positively correlated with PD-L1 expression and PD-1 immune checkpoint-related signaling pathways in nine patients with tongue squamous cell carcinoma (Figure 2G). In addition, RNA-seq was performed on WT and SPHK1-depleted SAS and SCC15 cells, and differential gene expression analysis showed that MMP1 expression was positively correlated with SPHK1 (Figure S2A). Analysis of TCGA database revealed a positive correlation between SPHK1 and MMP1 mRNA levels (Figure S2B). Immunofluorescence staining showed that SPHK1, MMP1, and PD-L1 co-localized identically in the HNSCC cell lines SAS and SCC15 (Figure 2I). By constructing stable HNSCC cell lines SAS and SCC15 with knocked-down SPHK1 through viral transfection, we discovered that knocking down SPHK1 reduced the mRNA and protein levels of MMP1 and PD-L1 in HNSCC (Figure 2J, K). In the MMP1-knockdown cell lines SAS and SCC15, knocking down MMP1 reduced the mRNA and protein levels of PD-L1 in HNSCC cells without affecting the mRNA and protein levels of SPHK1 (Figure 2J, K). In contrast, in virus transfected HNSCC cell lines SAS and SCC15 constructed with stable overexpression of SPHK1, overexpression of SPHK1 increased the protein levels of MMP1 and PD-L1 in HNSCC. Meanwhile, overexpression of MMP1 did not affect the protein level of SPHK1, but it did increase the protein level of PD-L1 (Figure S2C). Therefore, the above experimental evidence indicates that SPHK1 positively regulates PD-L1 expression in a MMP1-dependent manner in HNSCC.

### SPHK1-MMP1 enhances the ability of T cells to kill HNSCC cells *in vitro*

**Figure 3:**
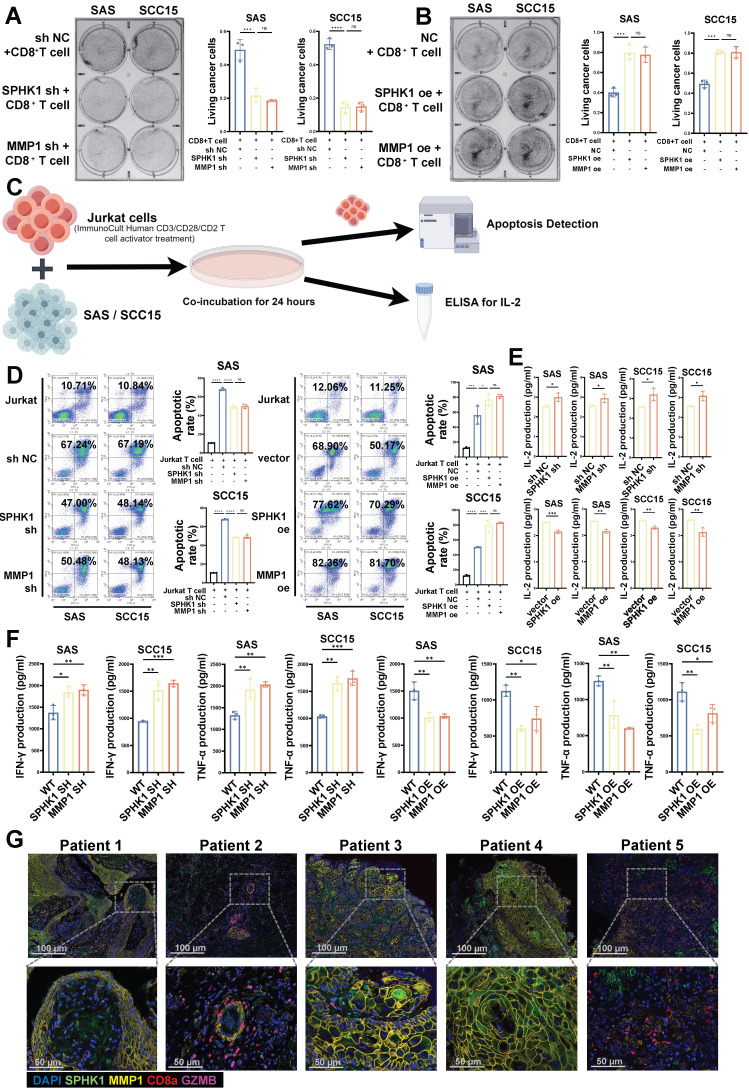
SPHK1 and MMP1 inhibited the ability of T cells to kill HNSCC *in vitro*. **A-B.** Effects of SPHK1 and MMP1 on cancer cell killing. The HNSCC cell lines SAS and SCC15 with knockdown/overexpression of SPHK1/MMP1 were co-cultured with activated human CD8^+^ T cells for 48 h to observe the effects of SPHK1 and MMP1 on T cells. The surviving cancer cells were stained with crystal violet. The ratio of cancer cells to T cells was 1:3. Representative images are shown on the left, and quantitative data are shown on the right. Schematic of activated Jurkat T cells co-cultured with SAS and SCC15 cells and subjected to an apoptosis assay and ELISA for IL-2 measurement. **C-D.** Effects of SPHK1 and MMP1 on the apoptosis of Jurkat cells co-cultured with tumor cells. After activated Jurkat cells were co-cultured with the HNSCC cell lines SAS and SCC15 for 24 h, apoptosis was detected by flow cytometry. The ratio of cancer cells to Jurkat cells was 1:5. Representative images are shown on the left, and quantitative data are shown on the right. Apoptosis of Jurkat cells cultured alone was used as a control.** E.** Effects of SPHK1 and MMP1 on IL-2 secretion by Jurkat cells. Histograms showing the levels of IL-2 secreted by Jurkat T cells co-cultured for 24 h with the HNSCC cell lines SAS and SCC15 with knocked down/overexpressed SPHK1/MMP1. The level of IL-2 secreted by Jurkat cells co-cultured with the HNSCC cell lines SAS and SCC15 without knockdown/overexpression was used as a control. **F.** SAS and SCC15 cells co-cultured with activated CD8^+^ T cells for 48h. IFN-γ and TNF-α levels measured by ELISA. **G.** Multiplex immunofluorescence staining of tumor tissues from five patients with HNSCC showed the ratio of SPHK1^+^/MMP1^+^ /CD8a^+^/GZMB^+^ cells. Error bars represent the mean ± SD of three independent experiments. Statistical significance was determined using one-way ANOVA with Tukey's multiple comparisons test. *P < 0.05; **P < 0.01; ***P < 0.001. NS: No significance, PD-L1: Programmed cell death ligand 1.

Binding of PD-L1 in tumor cells to PD-1 in CD8^+^ T cells inhibits the effector functions of T cells. Therefore, we conducted CD8^+^ T-cell-mediated tumor cell killing experiments, as previously described, to evaluate the effects of SPHK1 and MMP1 on the ability of T cells to kill cancer cells in HNSCC. The HNSCC cell lines SAS and SCC15 with knocked-down/overexpressed SPHK1/MMP1 were co-cultured with activated human CD8^+^ T cells for 48 h to observe the effects of SPHK1 and MMP1 on T cells. The results showed that compared to the NC group, knocking down SPHK1 and MMP1 made HNSCC cells more sensitive to CD8^+^ T-cell-mediated killing, whereas overexpression of SPHK1 and MMP1 increased the survival rate of HNSCC cells co-cultured with T cells, indicating that SPHK1 positively regulates PD-L1 via MMP1 to reduce the ability of CD8^+^ T cells to kill HNSCC cells (Figure 3A-B). We also evaluated the effects of SPHK1/MMP1 on the apoptosis of immortalized T cells, namely Jurkat cells, co-cultured with the HNSCC cell lines SAS and SCC15 using flow cytometry (Figure 3C). The results showed that co-culturing with HNSCC cells increased the apoptosis rate of Jurkat cells, which could be reduced by knocking down SPHK1 and MMP1, whereas overexpression of SPHK1 and MMP1 increased the apoptosis rate (Figure 3D). We further explored the effects of SPHK1/MMP1 on T-cell proliferation activity in HNSCC by co-culturing Jurkat cells with SAS and SCC15 HNSCC cell lines with knocked-down/overexpressed SPHK1/MMP1 for 24 h and then measuring the expression of IL2, a key signaling molecule for T-cell proliferation, in the culture supernatants. The results illustrated that knocking down SPHK1/MMP1 increased IL2 secretion, indicating enhanced T-cell proliferation, whereas overexpression of SPHK1/MMP1 decreased IL2 secretion, indicating reduced T-cell proliferation activity (Figure 3E). Next, activated CD8^+^ T cells were co-cultured with HNSCC in the presence of SPHK1/MMP1 knockdown or overexpression for 48 h, and SPHK1/MMP1 was found to be negatively correlated with the levels of IFN-γ and TNF-α released by CD8^+^ T cells (Figure 3F). Immunofluorescence staining of tumor tissues from five patients with HNSCC showed that the protein expression levels of SPHK1 and MMP1 were higher in patients 1, 3, and 4, and lower in patients 2 and 5, whereas the protein expression levels of CD8a and GZMB were lower in patients 1, 3, and 4, and higher in patients 2 and 5 (Figure 3G). In conclusion, SPHK1 inhibits the ability of T cells to kill HNSCC cells *in vitro* through MMP1.

### SPHK1 regulates MMP1 expression via the MAPK-ERK1/2 axis

**Figure 4:**
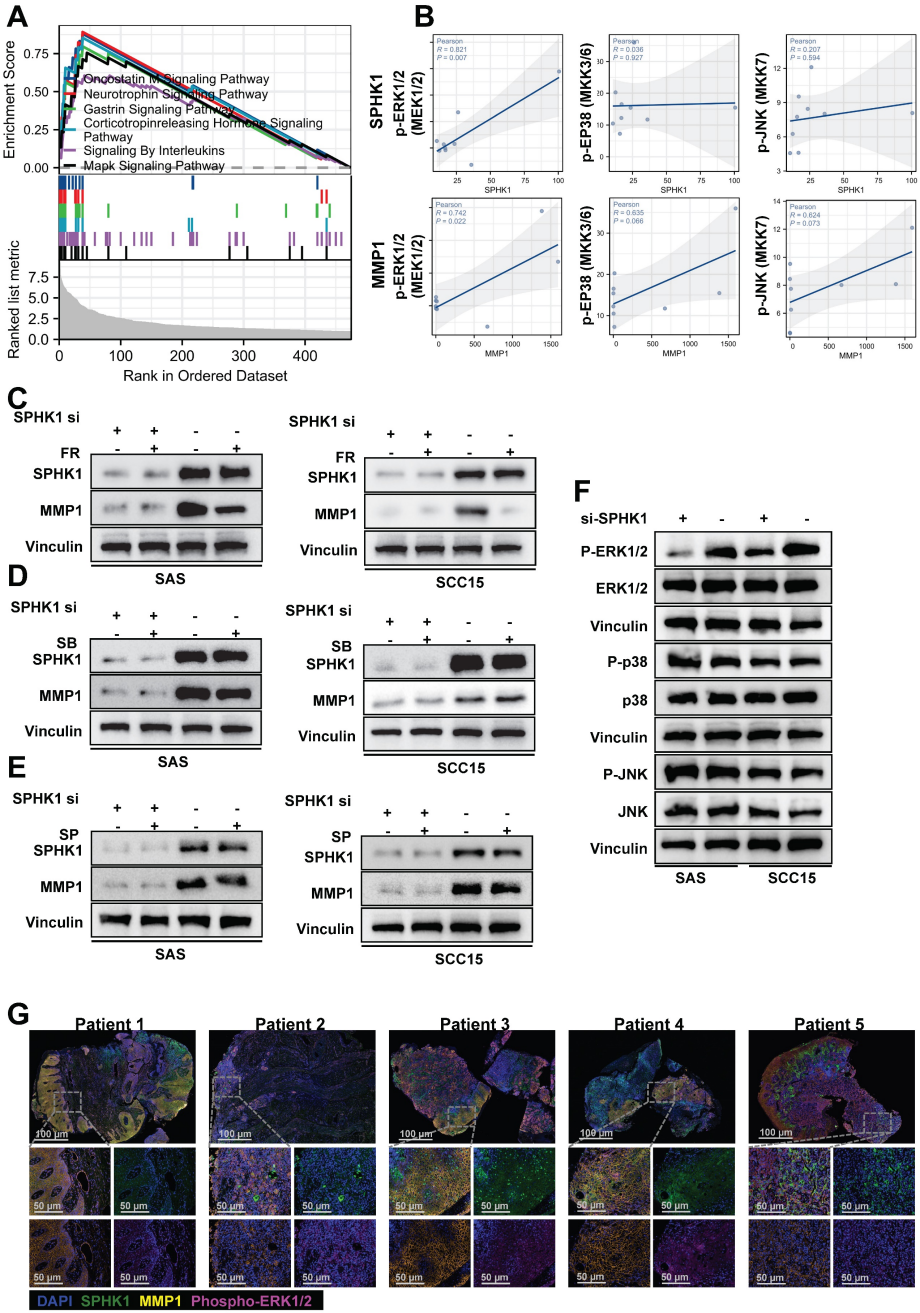
SPHK1 regulated MMP1 expression via the MAPK signaling pathway. **A.** Enrichment plot of SPHK1 target gene sets based on RNA sequencing. **B.** Correlation analysis of specific MAPK subfamily direct upstream kinases of ERK, p38, and JNK with SPHK1 and MMP1. **C-E.** SAS and SCC15 cells were treated with sphk1-specific siRNA or siRNA NC for 48 h, and then cells were transduced for 24 h in the presence or absence of 10 µM of the following inhibitors: ERK1/2 inhibitor FR 180204 (FR), JNK inhibitor SP 600125 (SP), or p38 inhibitor SB 202190 (SB). MMP1 protein levels were then analyzed by western blotting. **F.** SAS and SCC15 cells were treated with sphk1-specific siRNA or siRNA NC for 48 h and then analyzed for phosphorylated and total ERK1/2, phosphorylated and total p38, and phosphorylated and total JNK by western blotting. **G.** Multiplex immunofluorescence staining of tumor tissues from five patients with HNSCC showed the ratio of SPHK1^+^/MMP1^+^ /phospho-ERK^+^ cells.

Previous studies have indicated that members of the MAPK family are involved in the upregulation of MMP1 gene expression. To investigate whether MAPK family members are involved in the regulation of MMP1 by SPHK1, we performed gene set enrichment analysis (GSEA) of SPHK1 using RNA-seq data from fresh tumor tissues obtained from nine patients with tongue squamous cell carcinoma before immunotherapy. The analysis revealed significant enrichment of genes upregulated in the MAPK signaling pathway among downstream genes (Figure 4A). Correlation analysis of specific MAPK subfamily direct upstream kinases of ERK, p38, and JNK with SPHK1 and MMP1 revealed that SPHK1 and MMP1 were positively correlated with p-ERK1/2 (Figure 4B). The mechanism by which SPHK1 regulates MMP1 in the HNSCC cell lines SAS and SCC15 was verified using specific pharmacological inhibitors of ERK1/2, p38, and JNK. The results illustrated that the stimulation of MMP1 protein by SphK1 was reduced by the ERK1/2 inhibitor FR 180204, whereas the JNK inhibitor SP 600125 and the p38 inhibitor SB 202190 had no significant effect (Figure 4C, D, E). The ERK1/2 inhibitor FR 180204 also inhibited the increase in MMP1 and PD-L1 protein levels by SPHK1 overexpression (Figure S3A). Consistent with these findings, knocking down SPHK1 in SAS and SCC15 HNSCC cells inhibited ERK1/2 kinase activation but had no significant effect on JNK and p38 kinase activation (Figure 4F). Immunofluorescence staining of tissue samples from five patients with HNSCC revealed that patients 1, 3, and 4 had higher protein expression levels of SPHK1, MMP1, and p-ERK1/2, whereas patients 2 and 5 had lower protein expression levels of these markers (Figure 4G). In conclusion, these experiments demonstrate that the MAPK-ERK1/2 pathway is involved in the upregulation of MMP1 induced by SPHK1 in HNSCC.

### MMP1 forms a stable structure with PD-L1 through protein binding, and MMP1 can reverse the phenotype of SPHK1

**Figure 5:**
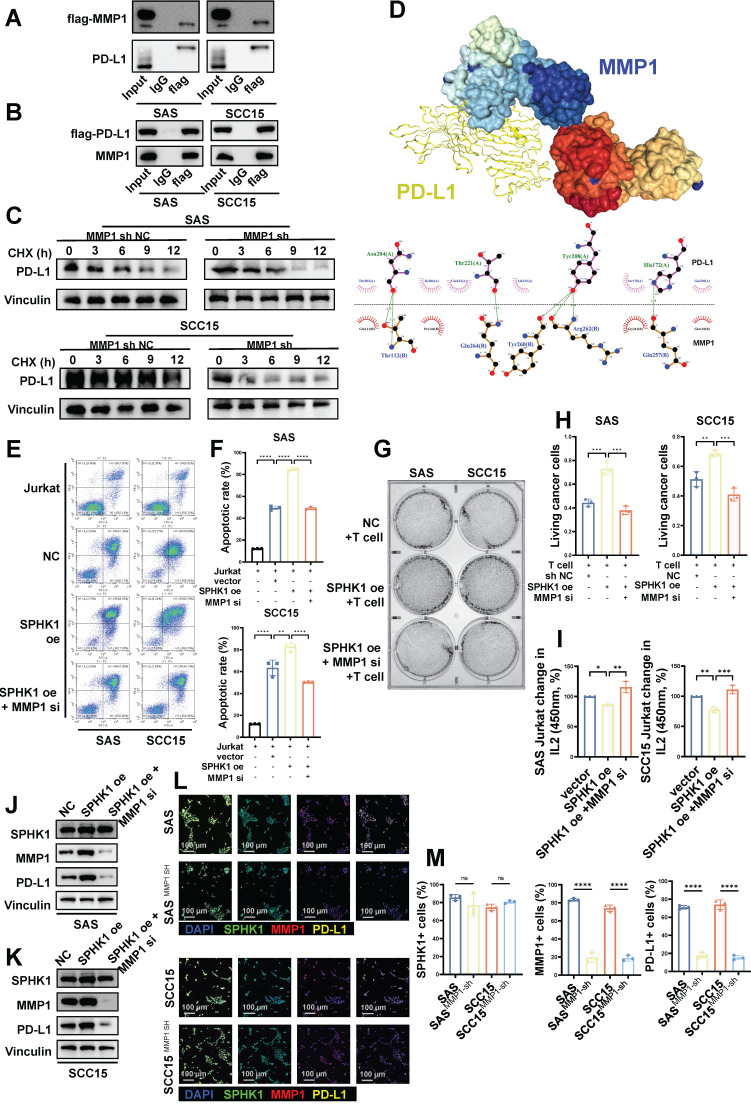
MMP1 formed a stable structure with PD-L1 in a protein-bound manner, and MMP1 reversed the SPHK1 phenotype. **A-C.** Co-IP showing the region of PD-L1 bound to MMP1. **A.** The total protein of SAS/SCC15 cells was immunoprecipitated with anti-MMP1 antibody, followed by immunoblotting with anti-PD-L1 antibody.** B.** The total protein of SAS/SCC15 cells was immunoprecipitated with anti-PD-L1 antibody, followed by immunoblotting with anti-MMP1 antibody. **C.** MMP1-related PD-L1 protein stability western blot assay. SAS and SCC15 cells from the protein translation blocker cycloheximide (CHX) treated (sh NC) control and MMP1 knockdown groups were used, and the rate of PD-L1 protein degradation was detected by western blot timing. **D.** Docking mode and interactions between MMP1 and PD-L1. **E-F.** Knockdown of MMP1 restored the effect of SPHK1 on apoptosis in Jurkat cells co-cultured with tumor cells. Apoptosis was detected by flow cytometry after activated Jurkat cells were co-cultured with MMP1-specific siRNA or siRNA NC-treated HNSCC cell lines SAS and SCC15 overexpressing SPHK1 for 24 h. The ratio of cancer cells to Jurkat cells was 1:5. Representative images are shown on the left, and quantitative data are shown on the right. Apoptosis of Jurkat cells cultured alone served as a control.** G-H.** Knockdown of MMPI rescued the ability of SPHK1 to inhibit HNSCC killing by T cells *in vitro*. The HNSCC cell lines SAS and SCC15 with SPHK1 overexpression were co-cultured with activated human T cells for 48 h after treatment with MMP1-specific siRNA or siRNA NC for 48 h, and the survival rate of cancer cells was determined. Staining of surviving cancer cells with crystal violet. The cancer cell to T cell ratio was 1:3. Representative images are shown on the left, and quantitative data are shown on the right. **I.** Knocking down MMP1 rescued the reduced IL2 secretion of T cells caused by SPHK1 overexpression. **J-K.** Knockdown of MMPI rescued SPHK1-induced high PD-L1 levels. After MMP1-specific siRNA or siRNA NC treatment of SPHK1-overexpressing HNSCC cell lines SAS and SCC15 for 48 h, PD-L protein levels were detected using western blotting. **L-M.** Multiplex immunofluorescence showed that MMP1 knockdown rescued the SPHK1-induced increase in the proportion of PD-L1^+^ cells. Error bars represent the mean ± SD of three independent experiments. Statistical significance was determined using one-way ANOVA with Tukey's multiple comparisons test. *P < 0.05; **P < 0.01; ***P < 0.001. NS: No significance, PD-L1: Programmed cell death ligand 1.

We next conducted co-immunoprecipitation experiments to investigate the mechanism by which MMP1 regulates PD-L1. The results illustrated that the interaction between Flag-tagged MMP1 and PD-L1 could be verified both positively and negatively in SCC15 and SAS cell lines (Figure 5A-B). Subsequently, we performed protein stability assays: after knocking down MMP1, we treated control and MMP1-depleted cells with the protein translation inhibitor cycloheximide (CHX) to observe the degradation rate of PD-L1. The results illustrated that the degradation rate of PD-L1 was significantly accelerated after knocking down MMP1 (Figure 5C). Lastly, we used pyMOL and Ligplot to visualize the molecular docking results for MMP1 and PD-L1. PD-L1 binds to the MMP1 target primarily through the formation of multiple hydrogen bonds and hydrophobic interactions with amino acid residues (Figure 5D).

Subsequently, we performed rescue experiments to verify whether MMP1 could reverse the changes in SPHK1-induced PD-L1 expression and T-cell function induced by SPHK1 in HNSCC. The HNSCC cell lines SAS and SCC15 with SPHK1 overexpression were co-cultured with Jurkat cells. The results demonstrated that knocking down MMP1 reduced the high apoptosis rate of Jurkat cells induced by SPHK1 (Figure 5E-F). Furthermore, when the HNSCC cell lines SAS and SCC15 with overexpression of SPHK1 were co-cultured with activated human T cells, knocking down MMP1 rescued the increased survival rate of HNSCC cells co-cultured with T cells induced by overexpression of SPHK1 (Figure 5G-H). Simultaneously, knocking down MMP1 also rescued the reduced IL2 secretion caused by SPHK1 overexpression (Figure 5I). By treating SAS and SCC15 cells with SPHK1 overexpression with MMP1 knockdown, it was discovered that MMP1 knockdown rescues the upregulation of PD-L1 protein levels induced by SPHK1 overexpression (Figure 5J-K). Immunofluorescence experiments revealed that knocking down MMP1 in SAS and SCC15 cells downregulated the proportion of PD-L1^+^ cells but did not affect the proportion of SPHK1^+^ cells (Figure 5L-M).

This section demonstrates the potential mechanism by which MMP1 influences PD-L1 expression in HNSCC: MMP1 forms a stable structure with PD-L1 through protein binding, inhibiting PD-L1 degradation and maintaining its expression. Additionally, the rescue experiments confirmed the upstream and downstream relationships within the regulatory axis of SPHK1/MMP1/PD-L1.

### In immune-competent mouse models, SPHK1 regulates PD-L1 expression via MMP1 and is associated with the efficacy of anti-PD-1 monoclonal antibodies

**Figure 6:**
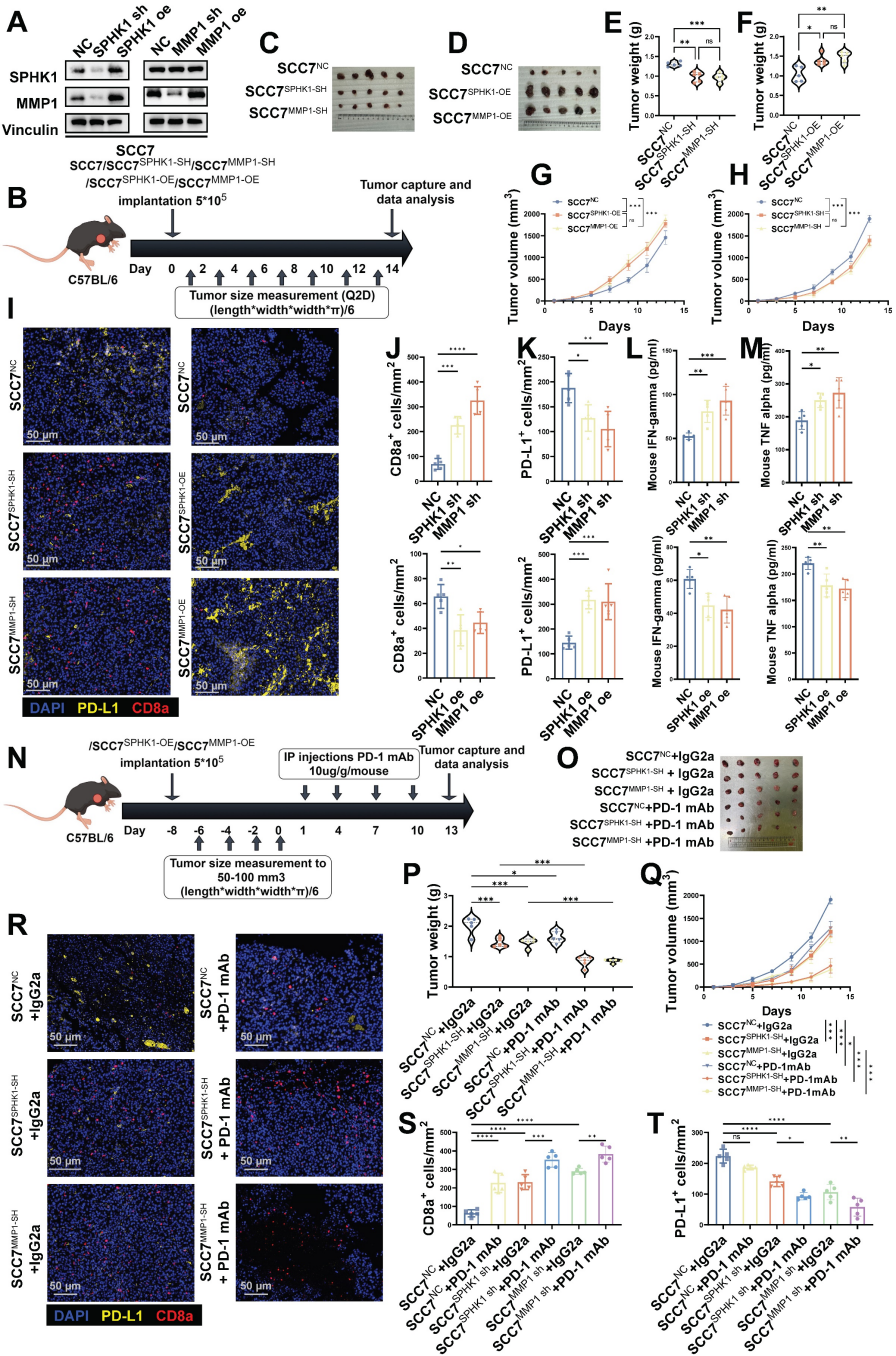
In the HNSCC immunocompetent mouse model, the SPHK1-MMP1 axis promoted tumor growth by promoting anti-tumor immunity. **A.** SCC7 HNSCC cells were transfected with either SPHK1, MMP1, or a negative control (NC) vector. Subsequently, the expression levels of SPHK1 and MMP1 were analyzed by western blotting.** B.** Schematic representation of the C57BL/6 female mouse model established via subcutaneous injection of SCC7 cells overexpressing/knocking down SPHK1, MMP1. Image of SCC7 tumors captured on day 14 (**C-D**). Tumor weight data harvested on day 14 (**E-F**). Plots of tumor volumes measured every 2 days (**G-H**). **I.** Tumors with knockdown or overexpression of SPHK1/MMP1 were subjected to PD-L1, CD8α dual-color immunofluorescence staining, and **J-K** shows quantitative data. **L-M.** IFN-γ and TNF-α ELISAs were performed on sera from HNSCC mouse models with knockdown or overexpression of SPHK1/MMP1. **N.** Schematic representation of the C57BL/6 female mouse model established by treating SCC7 cells knocked down with SPHK1 or MMP1 with PD-1 monoclonal antibody/IgG2a (100 μg/mouse). **O-P.** Tumor images and tumor weight data taken on day 13.** Q.** Plot of the tumor volume measured every 2 days. Dual-color immunofluorescence staining for PD-L1 and CD8α was performed on tumors treated with PD-1 mAb/IgG2α: **R** shows representative images, and **S-T** shows the quantitative data. Data represent mean ± SD. Statistical differences were determined using one-way analysis of variance. Significance levels are indicated as follows: *P < 0.05; **P < 0.01; ***P < 0.001. NS: No significance, PD-L1: Programmed cell death ligand 1.

To investigate the effects of SPHK1 and MMP1 expression on tumor biology, we initially generated SCC7 cells from HNSCC mice with normal immune function and stably overexpressed or knocked down SPHK1 or MMP1 (Figure 6A). Subsequently, these cell lines (SCC7, SCC7^SPHK1-OE^, SCC7^MMP1-OE^, SCC7^SPHK1-SH^, and SCC7^MMP1-SH^) were implanted subcutaneously into immunocompetent mice to induce tumor formation (Figure 6B). The results indicated that knocking down SPHK1/MMP1 reduced tumor growth rates in mice, whereas overexpression of SPHK1/MMP1 accelerated tumor growth (Figure 6C-H). After euthanizing the mice and harvesting the tumors, immunofluorescence staining revealed that SPHK1/MMP1 overexpression significantly enhanced PD-L1^+^ cell counts and decreased the density of CD8^+^ T cells within the tumor region. Conversely, knocking down SPHK1/MMP1 downregulated PD-L1^+^ cells and relatively increased the density of CD8^+^ T cells in the tumor area (Figure 6I-K). On the day of tumor capture, IFN-γ and TNF-α ELISAs were performed on sera from HNSCC mouse models with knockout or overexpression of SPHK1/MMP1, with the results showing that SPHK1/MMP1 expression was negatively correlated with IFN-γ and TNF-α (Figure 6L-M).

To study the effect of anti-PD-1 therapy on SPHK1- or MMP1-mediated immune evasion *in vivo*, we inoculated immunocompetent mice with the SCC7, SCC7^SPHK1-SH^, and SCC7^MMP1-SH^ cell lines. When the tumor size reached 50-100 mm^3^, PD-1 mAb/IgG2α was administered intraperitoneally simultaneously (Figure 6N).

Consistent with previous findings in mouse models, the knockdown of SPHK1/MMP1 significantly reduced tumor growth (Figure 6O-Q), increased intratumoral PD-L1^+^ cell density, and inhibited CD8^+^ T cell exhaustion (Figure 6R-T). In addition, the combination of SPHK1/MMP1 knockdown with anti-PD-1 therapy resulted in more pronounced tumor suppression. This combination therapy further alleviated CD8^+^ T-cell exhaustion (Figure 6R-T). In conclusion, the SPHK1-MMP1-PD-L1 regulatory axis was demonstrated in a mouse model and promoted the growth of HNSCC. The prospect of SPHK1/MMP1 knockdown combined with the anti-PD-1 therapeutic regimen represents an efficacious approach to enhance T cell-mediated anti-tumor immunity, offering a promising avenue for potential advancements in cancer therapy.

### Levels of SPHK1 and MMP1 are independent predictors of poor prognosis in HNSCC

**Figure 7:**
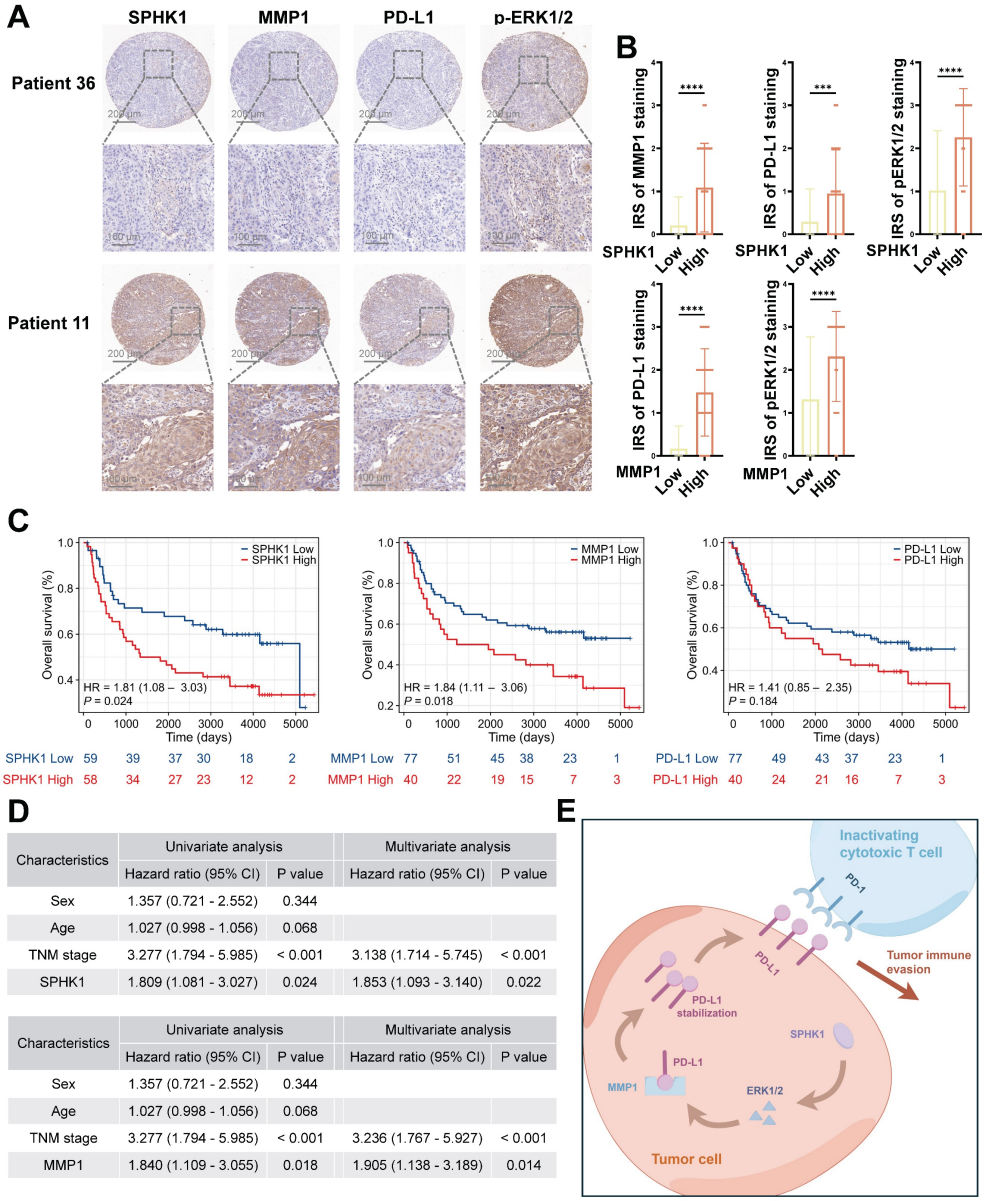
SPHK1 and MMP1 levels are independent predictors of unfavorable prognosis in HNSCC.** A.** SPHK1, MMP1, PD-L1, and pERK1/2 protein expression was evaluated via IHC staining in 117 HNSCC tumor tissues. Scale bars: 200 μm (upper panel), 100 μm (lower panel). **B.** IHC scores for four proteins in HNSCC tissues with high and low expression of SPHK1 or MMP1 (n = 117). The data are presented as the mean ± SD. Statistical analysis was performed using the two-tailed Mann-Whitney test. **C.** Kaplan-Meier analysis of overall survival based on SPHK1, MMP1, and PD-L1 expression levels. Statistical analysis was performed using the log-rank test.** D.** Tables showed the significance of different prognostic variables in the overall survival of patients with HNSCC. Statistical analysis was performed using multivariate Cox regression analysis. The data are presented as the mean ± SD. **E.** Schematic representation of the mechanism by which SPHK1 promotes tumor immune evasion in HNSCC via MMP1 regulation of PD-L1.

To elucidate the clinical significance of SPHK1 and MMP1 in HNSCC, we conducted immunohistochemical staining (Figure 7A) and immune reactivity scoring (IRS) analysis on 117 HNSCC specimens. We were thus able to confirm a positive correlation among SPHK1, MMP1, PD-L1, and pERK1/2 (Figure 7B). Furthermore, Kaplan-Meier analysis revealed that SPHK1 and MMP1 levels were negatively correlated with OS, as were the PD-L1 levels, although the difference did not reach statistical significance (Figure 7C). Multivariate Cox regression analysis identified SPHK1 and MMP1 expression as independent risk factors for HNSCC (Figure 7D). In conclusion, these clinical results indicate that the SPHK1-MMP1 axis regulates PD-L1 expression in HNSCC and that SPHK1 and MMP1 levels can predict the prognosis of HNSCC.

## Discussion

In the current study, we elucidated the molecular mechanism by which SPHK1 regulates tumor PD-L1 expression via MMP1. Additionally, pERK1/2 was identified as a crucial target of SPHK1 for regulating MMP1 and was found to be involved in the regulation of PD-L1 expression. Our findings also demonstrated that SPHK1-MMP1 maintains an immunosuppressive state in HNSCC and promotes tumor immune evasion by positively regulating PD-L1 expression in squamous cancer cells, reducing the proportion of TILs, and inhibiting the ability of T cells to kill cancer cells. Importantly, the combination of SPHK1/MMP1 knockdown and anti-PD-1 therapy produces more obvious tumor suppression and further alleviates CD8+ T-cell exhaustion. Therefore, SPHK1 and MMP1 are potential biomarkers for predicting the therapeutic response of patients with HNSCC to PD-1 mAb blockade therapy.

These findings are noteworthy for several reasons. First, we uncovered a novel and previously unidentified association among SPHK1, MMP1, and PD-L1, all of which are crucial molecules in tumor initiation and progression, particularly in highly immunogenic HNSCC. Additionally, the role of SPHK1 in cancer has garnered significant attention 36-37. Prior studies conducted by SubbaRao demonstrated that targeting SPHK1 could reduce pAkt expression and arrest cells in the G0/G1 phase of the melanoma cell cycle, thereby decreasing tumor cell proliferation and inducing apoptosis 37-38. Furthermore, research by Caroline Imbert et al. showed that combination strategies involving SPHK1 inhibition enhanced immune checkpoint blockade therapy by suppressing immunosuppressive cells in tumors 39-40. In line with these studies, we illuminated the role of SPHK1 in the initiation and progression of HNSCC, further filling a critical knowledge gap regarding SPHK1-mediated immune evasion in HNSCC. Moreover, MMP1 was identified for the first time as a potential target of SPHK1 and was found to be involved in the transcriptional regulation of PD-L1 in HNSCC.

As a member of the MMP family, MMP1 degrades various protein components in the extracellular matrix and regulates the migration, infiltration, and activation of immune cells 41-42. The biological characteristics of MMP1 in various cancers have been recognized 43-45; for instance, downregulation of MMP1 by BMP-6 can inhibit tumor metastasis in breast cancer 46.

In this study, we observed the expression of MMP1 in HNSCC cells and demonstrated that MMP1 promotes HNSCC progression *in vivo*. Importantly, the role of MMP1 in inhibiting the proportion of TILs and weakening the killing ability of T cells against cancer cells provides new insights for future research and clinical treatment of HNSCC.

In this study, we assessed the influence of SPHK1 and MMP1 on tumor immunity and observed that both SPHK1 and MMP1 inhibited the ability of T cells to kill cancer cells *in vitro*, while promoting HNSCC growth *in vivo* by facilitating tumor immune evasion in mice. Additionally, we found that SPHK1 and MMP1 levels correlated with the antitumor effects of PD-1 mAb in mice. A plausible explanation for this phenomenon is that MMP1 can form a stable structure with PD-L1 to inhibit its degradation. Previous research by Poyee L et al. showed that PD-1 mAb treatment for melanoma can rescue SPHK1-PD-L1-induced immune surveillance escape 47. Our research shows that the combination of SPHK1/MMP1 knockdown and anti-PD-1 therapy produces more obvious tumor suppression and further alleviates CD8^+^ T-cell exhaustion, which is consistent with previous research by Poyee et al.

Our observational results were also significant, as the SPHK1-MMP1 axis held potential value in predicting the prognosis of patients with HNSCC undergoing PD-1 inhibitor therapy. We speculated that the high expression of PD-L1 mediated by the SPHK1-MMP1 axis in HNSCC promoted adaptive immune resistance, which could have been rescued through PD-1 blockade. Prior studies emphasized the importance of PD-1^+^CD8^+^ T cells in the TME and revealed that a high percentage of the PD-1^+^CD8^+^ T cell subset was closely associated with the efficacy of ICIs 48. Furthermore, it has been confirmed that patients who respond to anti-PD-1 therapy have a higher PD-1^+^CD8^+^ T cell population compared to non-responders 49-50. Based on these findings, we believe that the existing immune suppression caused by the SPHK1-MMP1 axis could be reversed through anti-PD-1 mAb treatment, which manifests as the accumulation of tumor-infiltrating PD-1^+^CD8^+^ T cells.

We have elucidated a novel function of SPHK1 in HNSCC, established that SPHK1 regulates PD-L1-mediated immune suppression in HNSCC through MMP1, and revealed a novel mechanism of PD-L1 expression. Importantly, the SPHK1-MMP1 axis serves as an independent risk factor for predicting survival in patients with HNSCC and possesses the capability to modulate the response to PD-1 mAb therapy.

## Methods

### Clinical specimens

A total of 117 paraffin-embedded HNSCC specimens were used for survival analysis. These specimens were collected from the Pathology Department of Sun Yat-sen University Cancer Center between 2011 and 2022. All specimens represented post-surgical tumor tissue from the patients. The histopathological types of the tumors were classified according to the classification system established by the World Health Organization. Additionally, the tumor-node-metastasis staging was reclassified according to the 8th edition of the American Joint Committee on Cancer (AJCC) Cancer Staging Manual. The comprehensive clinical characteristics of the patients are presented in Table S1. This study was approved by the Institutional Ethics Review Committee of Sun Yat-sen University Cancer Center (B2022-698-01). The inclusion criteria for the 117 patients were as follows: (1) Patients aged 18 to 70 years; (2) patients were diagnosed with squamous cell carcinoma of the head and neck by histopathological examination, and with T1-T4a stage lesions by enhanced CT of the head and neck and electronic fiber laryngoscopy; (3) according to the AJCC staging criteria for HNSCC, all patients were clinically staged I to IV; (4) all patients completed chest enhanced CT, thyroid and cervical lymph node color Doppler ultrasound, electronic fiber gastroscopy, liver, gallbladder, pancreas, spleen, kidney color Doppler ultrasound, electrocardiogram, and other examinations; (5) the included patients had complete medical records; and (6) patients who completed surgical treatment in our hospital and retained postoperative pathology. The exclusion criteria were as follows: (1) Patients with multiple primary tumors in other parts of the body at the same time; (2) patients with recurrence or distant metastasis of HNSCC; (3) patients with poor function of major organs and inability to tolerate general anesthesia surgery; (4) patients with a history of other tumor diseases; (5) patients with central nervous system diseases or mental illnesses that lead to short- or long-term loss of self-awareness.

This study included nine patients with locally advanced HNSCC who were treated with PD-1 inhibitors as neoadjuvant therapy between January 1, 2019 and January 15, 2023 for transcriptomic analysis. Among them, eight were male and one was female, aged 38-70 years. This study was approved by the Ethics Committee of the Cancer Center of Sun Yat-sen University (approval number: B2023-481-01. Considering the retrospective nature of the study, the requirement for informed consent was waived. The inclusion criteria for the 117 patients were as follows: (1) Patients aged 18 to 70 years; (2) with confirmed squamous cell carcinoma of the head and neck region by histopathological examination, and confirmed to have T3-T4a lesions by enhanced CT of the head and neck and electronic fiber laryngoscopy; (3) all patients were clinically staged at stage III to IV according to the AJCC staging standard for HNSCC; (4) the major organ functions of the patients were normal, with hemoglobin ≥ 80 g/L, platelets ≥ 100 × 10^9^/L, white blood cells ≥ 3.5×10^9^/L, alanine aminotransferase ≤ 2.5× the upper limit of normal value, aspartate aminotransferase ≤ 2.5× the upper limit of normal value, urea nitrogen ≤ 1.25× the upper limit of normal value, and creatinine ≤ 1.25× the upper limit of normal value; (5) all patients completed chest enhanced CT, thyroid and cervical lymph node color Doppler ultrasound, electronic fiber gastroscopy, liver, gallbladder, pancreas, spleen, kidney color Doppler ultrasound, electrocardiogram and other examinations; and (6) the medical records of the selected patients were complete. The exclusion criteria were as follows: (1) Patients with a history of chemotherapy, radiotherapy, concurrent chemoradiotherapy, targeted therapy, head and neck surgery, or immunotherapy; (2) patients with multiple primary tumors in other parts of the body at the same time; (3) patients with recurrent or distant metastatic HNSCC; (4) patients with hypopharyngeal cancer/larynx whose primary lesion was assessed as amenable to laryngeal preservation surgery before treatment; (5) patients with major organ dysfunction who could not tolerate general anesthesia; (6) patients with a history of other tumor diseases; and (7) patients with central nervous system diseases or mental illnesses that resulted in short- or long-term loss of self-awareness.

Efficacy evaluation after neoadjuvant therapy: 2 weeks after the end of neoadjuvant therapy, patients underwent enhanced head and neck CT, electronic fiber laryngoscope, and biopsy pathology to evaluate efficacy. The clinical neoadjuvant efficacy was based on the latest version of the solid tumor treatment efficacy evaluation criteria, i.e., RECIST 1.1. Complete remission (CR) was defined as the disappearance of all target lesions, with no new lesions appearing, and the duration lasts for at least 4 weeks; partial remission (PR) was defined as the sum of the maximum diameters of target lesions decreasing by ≥ 30%, and the duration lasting for at least 4 weeks; stable disease (SD) was defined as the sum of the maximum diameters of target lesions decreasing but not reaching PR, or the sum of the maximum diameters of target lesions increasing but not reaching progressive disease (PD); and PD was defined as the sum of the maximum diameters of target lesions increasing by ≥ 20%, or the appearance of new lesions. CR and PR are considered indicative of effective neoadjuvant therapy, and SD and PD are considered indicative of ineffective neoadjuvant therapy.

### Animal experiments

The animal experiments in this study were approved by the Experimental Animal Ethics Committee of Sun Yat-sen University Cancer Center (L102042023120C). Six-week-old female C57BL/6 mice were purchased from the Guangdong Medical Laboratory Animal Center (Foshan, China) and subsequently housed in a controlled environment at the Animal Experiment Center of Sun Yat-sen University to ensure specific pathogen-free conditions. C57BL/6 mice were subcutaneously injected with 1 × 10^6^ SCC7cells of NC, SPHK1-OE, MMP1-OE, SPHK1-SH and MMP1-SH, and euthanized on day 21. For mice requiring PD-1 mAb (100 μg per mouse, BioXcell, BE0146), treatment was initiated when the tumor size reached 50-100 mm^3^ via intraperitoneal administration every other day for a total of four doses. Mice were euthanized for tumor excision 2 days after the final injection. Tumor volume changes and excised tumor weights were recorded for all mice.

### Cell Lines

The HNSCC cell lines SCC15, SAS, and SCC7 and the human normal oral epithelial keratinocyte line HOK were provided by Professor Wu Di at Sun Yat-sen University Cancer Center (Guangzhou). The human T-lymphoblastic leukemia cell line Jurkat was purchased from the American Type Culture Collection (ATCC). All cell lines were maintained in RPMI-1640 (Corning, 10-040-CVRC) or DMEM (Corning, 10-013-CVRC) supplemented with 10% fetal bovine serum (FBS; ExCell Bio, FSP500) and 100 U/ml penicillin-streptomycin (Gibco, 15140122).

### Lentiviral-mediated gene transfer

SAS and SCC15 cells were co-transfected with pSPAX2, pMD2G, and pSIN-EF2-puro vectors encoding SPHK1 and MMP1, respectively. Following an 8-h incubation period after transfection, the cell culture medium was replaced with Ultra-culture medium (Lonza, 12-725F). After a 48-h incubation period, the viral supernatant was collected, titrated, and used to infect the designated cells overnight. To confirm successful infection, puromycin (2 μg/ml, Solarbio, P8230) was administered.

### Short interfering RNA transfer

Short interfering RNA (siRNA) molecules were designed according to (Table S2) and were synthesized by GenePharma, Shanghai, China. Transfection of siRNAs was performed using Lipofectamine@3000 (Invitrogen, L3000015) following the manufacturer's instructions.

### Western blotting

Proteins were extracted from cells using RIPA lysis buffer (Byotime, P0013) supplemented with protease and phosphatase inhibitors (Byotime, P1045). Proteins were separated by SDS-polyacrylamide gel electrophoresis and then transferred onto NC membranes (Merck Millipore, HATF00010). The membranes were blocked with 5% bovine serum albumin and incubated with primary antibodies. Subsequently, peroxidase-conjugated secondary antibodies were applied to the membranes. The antigen-antibody interactions were visualized using an enhanced chemiluminescence assay (ECL, Thermo, 32106). The antibodies used are listed in Table S3. The results of repeated WB experiments are shown in the supplementary material (Repeated Verification of Western Blot and Gray Value Statistics).

### RNA extraction and RT-qPCR

RNA extraction was performed using the AllPrep RNA/DNA Mini Kit (Qiagen, 80204) according to the manufacturer's instructions. Complementary DNA (cDNA) was synthesized using random primers and M-MLV Reverse Transcriptase (Promega, M1705). qRT-PCR assays were conducted on the CFX96 Touch Real-Time PCR Detection System (Bio-Rad) or LightCycler 480 System (Roche) using ChamQ SYBR qPCR Master Mix (Vazyme, Q311-03). Data were normalized to GAPDH expression. The primer sequences are listed in Table S2.

### IHC staining and scoring

Paraffin-embedded tissue samples were cut into 3-μm-thick sections. Samples were incubated overnight at 4°C with primary antibodies against SPHK1 (1:200), MMP1 (1:200), PD-L1 (1:200), CD8a (1:200), and GZMB (1:200). The following day, immunodetection was performed using DAB (Dako, K5007) according to the manufacturer's instructions. The stained samples were imaged using an AxioVision Rel.4.6 computer image analysis system (Carl Zeiss). Immunohistochemical staining for SPHK1, MMP1, PD-L1, and pERK1/2 was scored independently by two experienced pathologists using the IRS system to ensure consistency and accuracy. The intensity of staining was graded on a scale of 0 (no brown particle staining), 1 (light brown particles), 2 (moderate brown particles), and 3 (dark brown particles). The proportion of positive tumor cells was categorized into four grades: 1 (< 10% positive cells), 2 (10%-40% positive cells), 3 (40%-70% positive cells), and 4 (> 70% positive cells). The IRS was calculated by multiplying these two grades, yielding a score ranging from (0-12)/4. High expression levels were defined as an IRS score ≥ 2, while low expression levels were defined as an IRS score < 2. The proportions of SPHK1, MMP1, PD-L1, and pERK1/2-positive cells were measured using HALO version 3.2.1851 (Indica Labs). The specific antibodies used for immunohistochemical analysis are listed in Table S3.

### Immunofluorescence staining

For immunofluorescence staining, cells were seeded onto chamber slides (Millipore) and fixed with 4% paraformaldehyde. Subsequently, cells were permeabilized with 0.25% Triton X-100 for 20 min and incubated with antibodies against SPHK1, MMP1, PD-L1, and other proteins. Subsequently, cells were incubated with DyLight® 594 anti-rabbit IgG (Vector Laboratories, DI-1794). The nuclei were counterstained with DAPI. Images were captured using an inverted confocal fluorescence microscope (LEICA TCS SP8). Immunofluorescence staining of tissue sections was performed as previously described. Four-micron frozen sections were fixed with 4% paraformaldehyde for 20 min at room temperature and permeabilized with 0.25% Triton X-100 for 20 min. Sections were then blocked with 5% bovine serum albumin for 30 min at room temperature. Sections were incubated with primary antibodies overnight at 4°C in blocking solution. Corresponding primary antibodies were used. For mouse antibodies, DyLight® 488 anti-mouse IgG (Vector Laboratories, DI-2788) was used; for rabbit antibodies, DyLight® 594 anti-rabbit IgG (Vector Laboratories, DI-1794) was used. The nuclei were counterstained with DAPI. Images were captured using an inverted confocal fluorescence microscope (LEICA TCS SP8) and analyzed using ImageJ software.

### CD8^+^T cell-mediated tumor cell killing assay

A T cell-mediated tumor cell killing assay was performed to investigate the impact of SPHK1/MMP1 on tumor immune evasion. According to the manufacturer's instructions, human CD8^+^ T cells were isolated from previously frozen peripheral blood mononuclear cells using a CD8^+^ T cell isolation kit (Stem Cell, 17953) and cultured in ImmunoCultTM -XF T cell Expansion Medium (Stem Cell, 10981) containing IL-2 (10 ng/ml, PeproTech, 200-02-50). ImmunoCultTM Human CD3/CD28 T Cell Activator (Stem Cell, 10991) was added to the cell suspension to activate T cells. The ratio of activated CD8^+^T cells to cancer cells was 3:1. Supernatants containing T cells and cellular debris were collected, and surviving cancer cells in the wells were washed with PBS, stained with crystal violet, and quantified using a spectrophotometer at an optical density of 570 nm. According to the manufacturer's instructions, the production of cytokines (IFN-γ and TNF-α) in the supernatant was quantified using ELISA kits (Biolegend, 430104 and 430204).

### Apoptosis and viability analysis of Jurkat cells co-cultured with cancer cells

To obtain activated Jurkat cells, Jurkat cells were cultured in RPMI 1640 medium containing 10% fetal bovine serum (FBS) and ImmunoCult Human CD3/CD28/CD2 T Cell Activator (STEMCELL Technologies, 10970) for 24 h. Human cancer cells were incubated in 6-well plates overnight before being co-cultured with activated Jurkat cells for 48 h. The ratio of activated Jurkat cells to cancer cells was 5:1. Jurkat cells were collected from the supernatants, and apoptosis was determined using the PE-Annexin V/7-AAD Kit (BD Biosciences, 559763) according to the manufacturer's instructions. The level of IL-2 secreted by Jurkat cells in the supernatants was detected by ELISA following the instructions of the Human IL-2 ELISA Kit (ExCell Bio, EH002).

### Bioinformatics analysis

To explore the correlation between the target gene and immune checkpoints, we analyzed the data from The Cancer Genome Atlas (TCGA) database. First, relevant data on the required cancer type, including gene expression data and clinical information, were downloaded from TCGA. The data were preprocessed to remove low-quality data and outliers to ensure data reliability and accuracy. Then, the expression data for the target gene and the expression data of known immune checkpoint-related genes were extracted. Appropriate statistical analysis methods were used to calculate the correlation coefficient between the expression of the target gene and each immune checkpoint gene, such as Pearson's correlation coefficient or Spearman's correlation coefficient. Through significance testing of the correlation coefficient, it is then determined whether there is a significant correlation between the target gene and immune checkpoints.

To analyze immune infiltration in the research samples, we adopted the core algorithm based on CIBERSORT. Specifically, the markers of 22 types of immune cells provided by the CIBERSORTx website (https://cibersortx.stanford.edu/) were used. First, the data to be analyzed were uploaded to this website. CIBERSORTx processes the uploaded data based on its computing power and specific algorithm processing. By identifying specific gene expression patterns in the data and comparing them with the known markers of 22 types of immune cells, the relative proportions of various immune cells in the sample were calculated to determine immune infiltration.

### Data processing of single-cell RNA-seq libraries and clustering

We analyzed a public single-cell dataset (GSE172577; www.ncbi.nlm.nih.gov/geo) comprising primary tumor tissues from six untreated patients with oral squamous cell carcinoma. The scRNA-seq reads were aligned to the GRCh38 reference genome and quantified using CellrangerCount version 2.2.0 (10x Genomics, Pleasanton, CA). Downstream analyses were performed using Seurat version 3.1.561. For human samples, cells meeting any of the following conditions were excluded from analysis: (i) > 20% mitochondrial RNA content; (ii) 200 genes detected; or (iii) > 40,000 UMI and 6000 genes detected. For NOG.EXL mouse samples, cells with (i) > 10% mitochondrial RNA content, (ii) 100 genes detected, or (iii) > 7000 genes detected were excluded from analysis.

For clustering all cell types, Seurat alignment was used across patients. The Seurat object containing all the cells was initially split by patient ID. Then, for each patient's object, raw UMI counts were log-normalized and variable genes were independently identified based on an average expression > 0.1 and an average dispersion > 1. Sets of anchors across patient objects were determined using the parameters CCA dims = 1:30 and the number of neighbors k.filter = 200, followed by integration. Scaled z-scores for each gene were then computed using the ScaleData function and regressed against the number of UMIs per cell and mitochondrial RNA content. Scaled data were used as input for principal component analysis of variable genes. Clusters were identified using shared nearest neighbor (SNN)-based clustering based on the top 83 significant principal components determined by the JackStraw function, with a resolution of 0.6 determined by the clustree output. The same principal components were used to generate UMAP projections, which were generated with a minimum distance of 0.3 and 30 neighbors. Differentially expressed (DE) genes for each identity cluster were generated using FindAllMarkers with min.pct = 0.25 and logfc.threshold = 0.25. Cell types were annotated using the resulting DE genes along with the expression of known marker genes.

For malignant cell clustering, subsets of cells that were identified as malignant based on broad clustering were isolated from the complete dataset. Then, the cells were clustered using Seurat without patient alignment because tumor cells are typically patient-specific. Raw UMI counts were normalized using SCTransform function with regularized negative binomial regression, where the cellular sequencing depth was used as a covariate in a generalized linear model. The number of UMIs per cell and mitochondrial RNA content were regressed out in a second non-regularized linear regression. Malignant cell clusters were identified using SNN-based clustering of the first 15 principal components with a resolution of 0.5. To assign the epithelial-mesenchymal transition score, the AddModuleScore function was used based on genes annotated with the GSEA MSigDB geneset “HALLMARK_EPITHELIAL_MESENCHYMAL_TRANSITION”.

### Quantification and statistical analysis

All experiments were repeated at least three times to ensure their reproducibility. The results are presented as the mean ± standard deviation (mean ± SD) and were analyzed using appropriate statistical methods according to the suitability of each method. Specifically, two-tailed Student's t-test, two-way analysis of variance (ANOVA), or one-way ANOVA combined with Tukey's multiple comparison test were employed. A P-value < 0.05 was considered statistically significant. Clinical characteristics were compared using the chi-square (χ^2^) test. Survival curves were then generated using the Kaplan-Meier method, and differences between groups were compared using the log-rank test to assess significance. To identify independent prognostic factors, multivariate analysis was performed using the Cox proportional hazards regression model. All statistical analyses were conducted using SPSS 27.0 statistical software, and GraphPad Prism 9.0 software was used for data analysis and visualization.

## Supplementary Material

Supplementary figures and tables.

## Abbreviations

ICIs: immune checkpoint inhibitors
HNSCC: head and neck squamous cell carcinoma
ORR: overall response rate
SPHKs: sphingosine kinases
TME: tumor microenvironment
PD-L1: programmed cell death ligand 1
PD-1 mAb: programmed cell death 1 monoclonal antibody
SPH: phosphorylation of sphingosine
MMPs: matrix metalloproteinases
MMP1: Matrix metalloproteinase-1
BMP-6: bone morphogenetic protein 6
MAPK: mitogen-activated protein kinase
TILs: tumor-infiltrating lymphocytes
TCGA: The Cancer Genome Atlas
RNA-seq: RNA sequencing
GSEA: gene set enrichment analysis
CR: complete remission
PR: partial remission
SD: stable disease
PD: progressive disease
ATCC: American type culture collection
siRNA: short interfering RNA
FBS: fetal bovine serum
SNN: shared nearest neighbor
