# Supplementary material for: SPHK1 promotes HNSCC immune evasion by regulating the MMP1-PD-L1 axis
Source: Theranostics. 2024 Oct 28;14(18):7199–218. doi: 10.7150/thno.102390 (PMC11610131; doi:10.7150/thno.102390)
Supplement: Supplementary file 1 — Supplementary figures and tables. [file thnov14p7199s1.pdf]

Figure S1

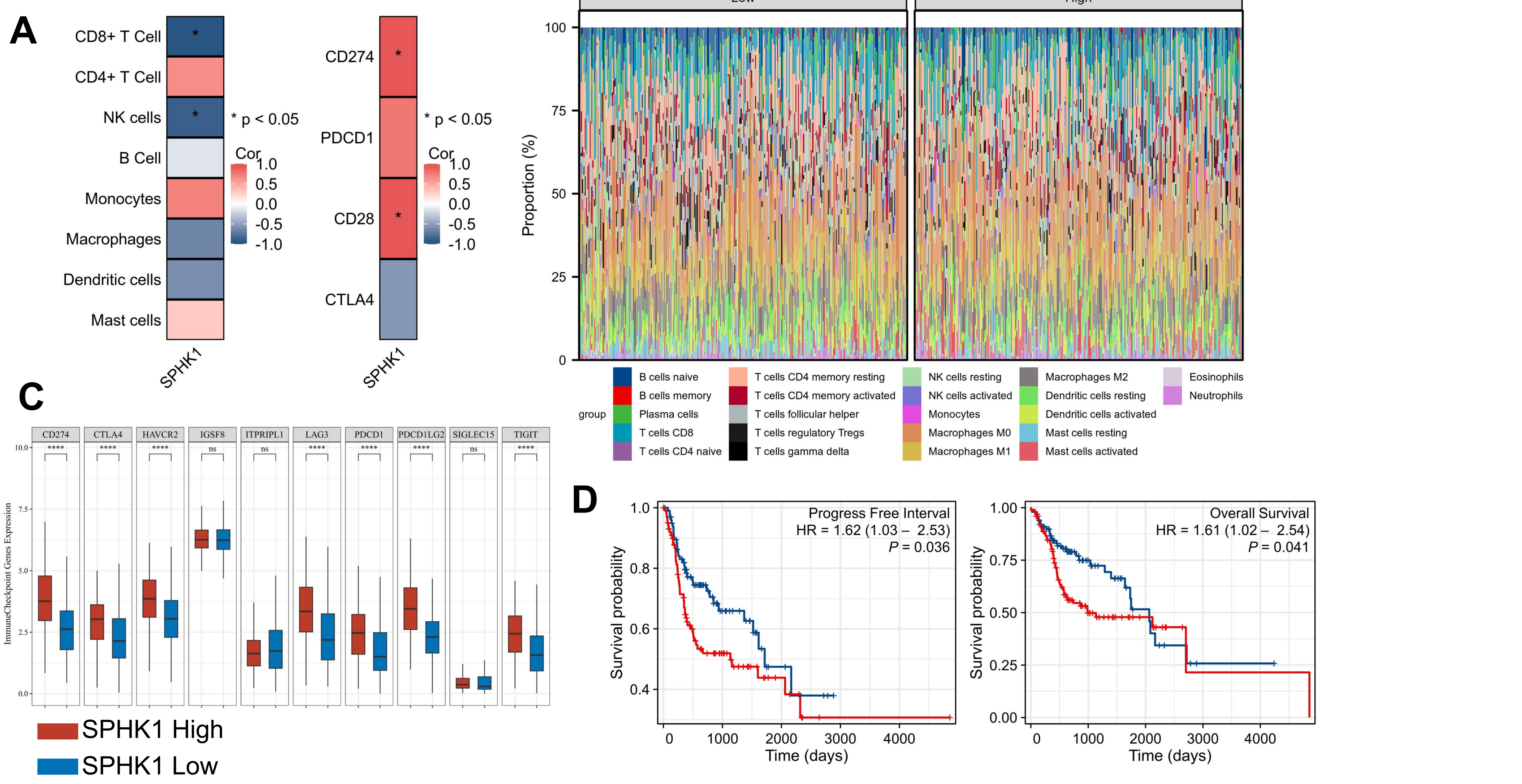

Figure S1. A. Correlation between the proportion of immune-infiltrating cells and immune checkpoint inhibitor levels with the level of SPHK1 in six patients with oral squamous carcinoma. **B-C.** Correlation between SPHK1 and immune infiltrating cells in HNSCC patients analyzed by TCGA data. C. Correlation between SPHK1 and immune checkpoint levels in HNSCC patients analyzed by TCGA data. **D.** Overall survival , progression-free survival Kaplan-Meier analysis based on SPHK1 expression level in HNSCC in TCGA database. Statistical analysis was performed using the log-rank test.

Figure S2

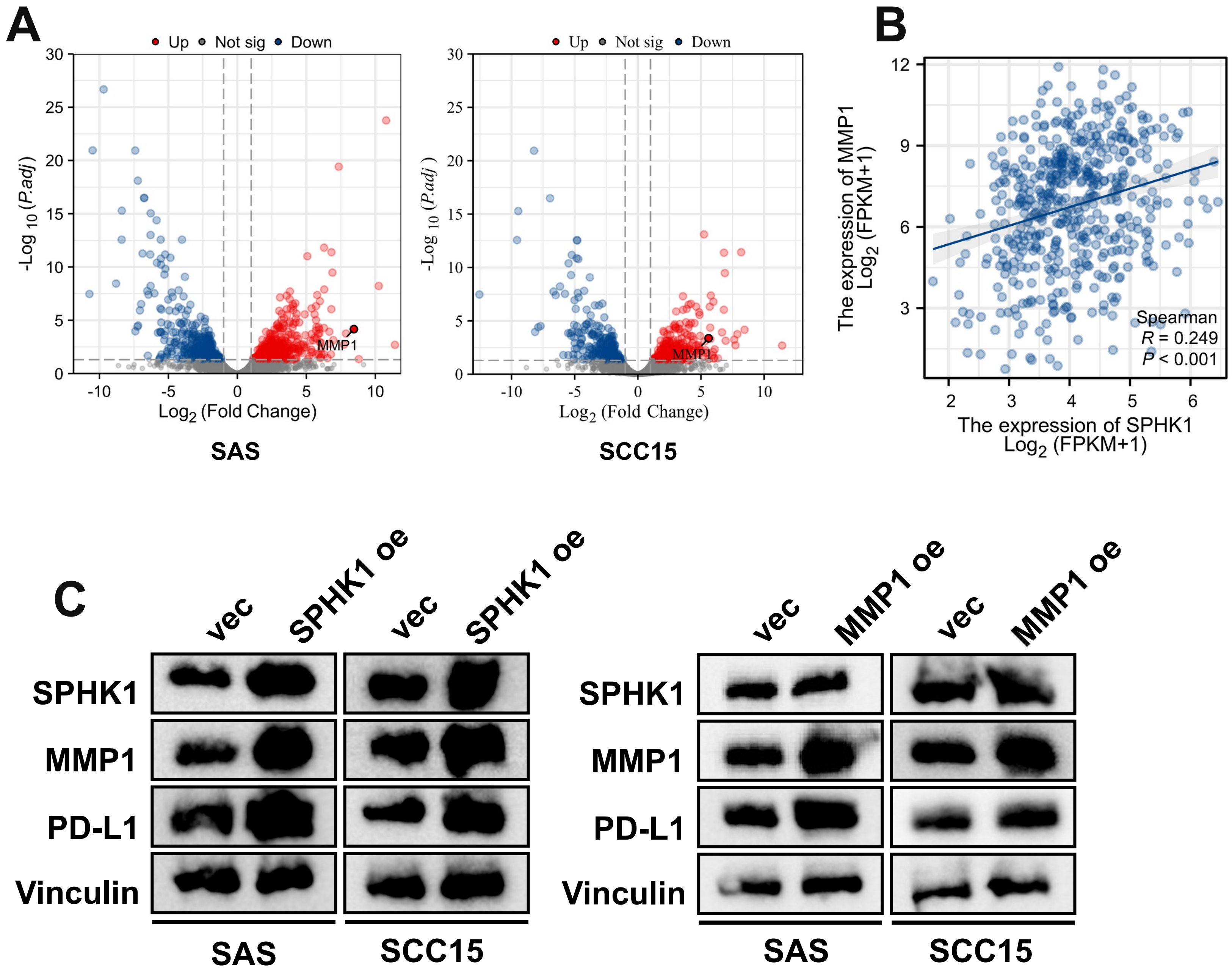

Figure S2. A. RNA-seq analysis and differential analysis were performed on WT and SPHK1-depleted SAS and SCC15 cells. **B.** Correlation analysis of SPHK1 levels with MMP1 in HNSCC patients in the TCGA database. **C.** Western blotting showed the effect of SPHK1/MMP1 overexpression in the HNSCC cell lines SAS and SCC15 on MMP1 and PD-L1 protein levels.

Figure S3

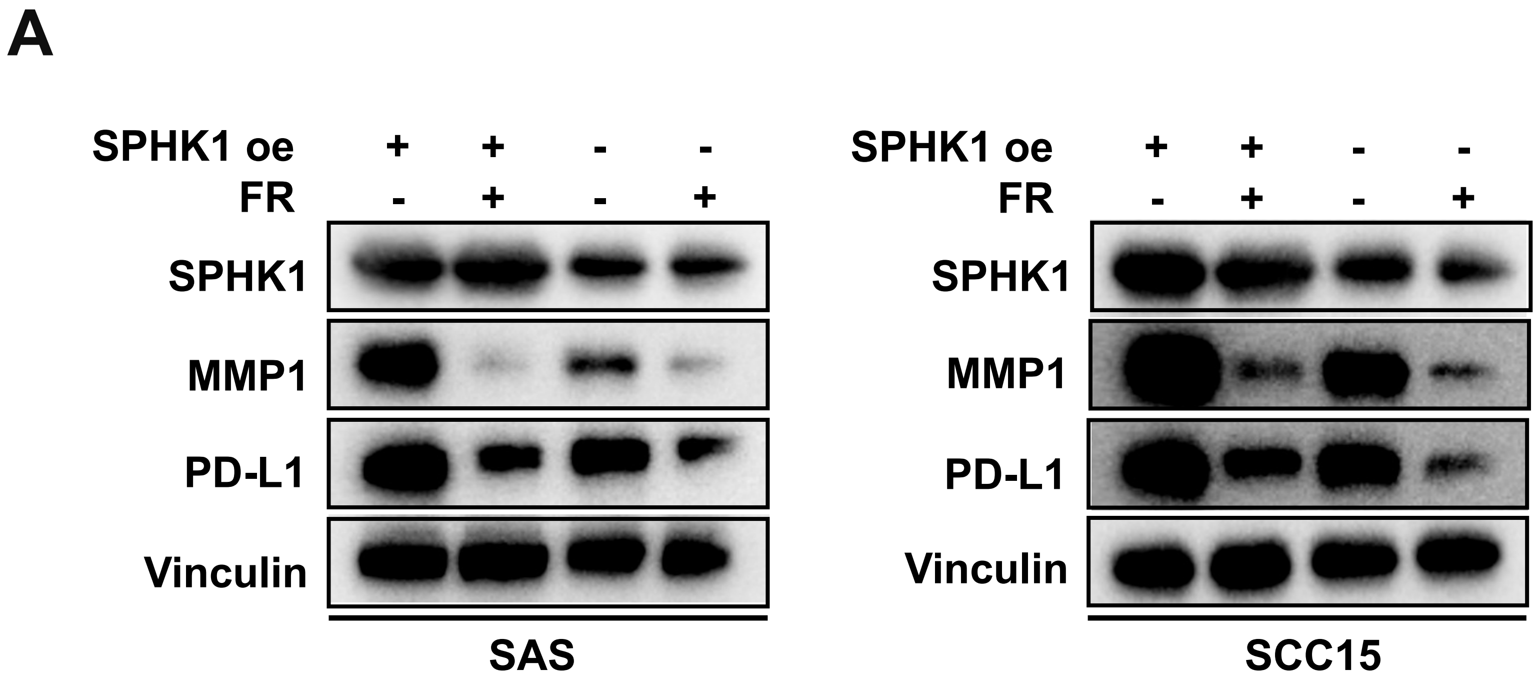

Figure S3. Western blotting showed the effect of ERK1/2 inhibitor FR 180204 on SPHK1 overexpression.

Supplementary Table 1. Baseline table of 117 HNSCC patients.

**Characteristic**	**Low expression group (SPHK1) n = 59 (100%)**	**High expression group (SPHK1) n = 58 (100%)**
Age		
≤47	6	3
＞47	53	55
Gender		
Male	42	47
Female	17	11
TNM stage		
I-II	31	17
III	8	19
IV	20	22

Supplementary Table 2. List of Oligonucleotides used in this study.

**Gene**	**Sense**	**Antisense**
**RT-qPCR primers**		
SPHK1	TCTATAGTGCTGTTGTCACACC	CAAGCTCCTCCGGCTACTGAGTC
GAPDH	GCGAACTCTGACTCTATCAGCG	ACCACCCTGTTGCTGTAGCCAA
MMP1	GCCTCTATTCATAGAGCGAAGC	TGCATCCCAGCGGCTGACGTGC
PD-L1	AGTAATCTATCAGCCTCTCAGT	CCTGTCGATGGCGAGCTTCTAGG
**siRNA targeting sequence**		
siSPHK1	GAGGCUGAAAUCUCCUUCATT	UGAAGGAGAUUUCAGCCUCTT
siMMP1	GCAGCUUCCUUGAACCAUUTT	AAUGGUUCAAGGAAGCUGCTT
siPD-L1	GUGCACCCAAACUACUUCUTT	AGAAGUAGUUUGGGUGCACTT

Supplementary Table 3. List of antibodies used in this study.

**Antibodies used for western blot (WB), Immunofluorescence (IF) and immunohistochemistry (IHC)**						
**anti-SPHK1**	**Supplier**	**Catalogue No.**	**Application**	**Host species**	**Species activity**	**Dilution**
anti-SPHK1	Abcam	ab26269	WB, IF, IHC	Rabbit	Hu, Mo	1:2000 for WB, 1:200 for IF, 1:200 for IHC
anti-SPHK1	Affinity Biosciences	DF6005-50	WB, IF	Mouse	Hu, Mo	1:1000 for WB
anti-MMP1	Abcam	ab52631-100ul	IF, IHC	Rabbit	Hu, Mo	1:100 for IF, 1:200 for IHC
anti-MMP1	Affinity Biosciences	DF6325-50	WB	Mouse	Hu	1:2000 for WB
anti-MMP1	ABclonal	A22080-50 µL	WB	Rabbit	Hu, Mo	1:10000 for WB
anti-CD274	Proteintech	28076-1-AP-50ul	IF, IHC	Rabbit	Hu, Mo	1:100 for IF, 1:200 for IHC
anti-CD274	ABclonal	A18103-50 µL	WB	Rabbit	Hu, Mo	1:3000 for WB
anti-CD274	ABclonal	A21443-50 µL	WB	Rabbit	Hu, Mo	1:2000 for WB
anti-CD8a	Servicebio	GB15068-100	IF	Mouse	Mo	1:100 for IF, 1:200 for IHC
anti-CD8a	Proteintech	CL594-66868	IF, IHC	Mouse	Hu	1:100 for IF
anti-GZMB	Proteintech	13588-1-AP-50ul	IF, IHC	Rabbit	Hu, Mo	1:100 for IF, 1:200 for IHC
anti-pERK1/2	Proteintech	51068-1-AP-50ul	IF, IHC	Rabbit	Hu, Mo	1:100 for IF, 1:200 for IHC
